# Death from Chloroform

**Published:** 1849-05

**Authors:** 


### DEATH FROM CHLOROFORM.

We have to record another death from chloroform. The Medical Times, of Feb. 17th, mentions the case. A young man in the Hotel Dieu of Lyons inhaled the vapor for a short time preparatory to having an operation performed. Before the operation was over life had become extinct. The effect of such a multiplication of these fatal instances must be to limit very much the use of this powerful agent.

